# Immune Persistence against SARS-CoV-2 after Primary and Booster Immunization in Humans: A Large-Scale Prospective Cohort Study

**DOI:** 10.3390/vaccines10101677

**Published:** 2022-10-08

**Authors:** Juan Li, Hui Xie, Weixin Chen, Meng Chen, Shuang Bai, Wei Zhao, Tao Zhou, Pei Gao, Lichi Zhang, Quanyi Wang, Xinghuo Pang, Chun Huang, Jiang Wu

**Affiliations:** Beijing Center for Disease Prevention and Control, Beijing 100013, China

**Keywords:** COVID-19, inactive vaccine, booster, Delta, Omicron, immune response

## Abstract

Amid the ongoing global COVID-19 pandemic, limited literature exists on immune persistence after primary immunization and the immunogenic features of booster vaccines administered at different time intervals. Therefore, this study aimed to determine the immune attenuation of neutralizing antibodies against the SARS-CoV-2 wild-type strain, and Delta and Omicron variants 12 months after the primary administration of the COVID-19 inactivated vaccine and evaluate the immune response after a booster administration at different time intervals. A total of 514 individuals were followed up after primary immunization and were vaccinated with a booster. Neutralizing antibodies against the wild-type strain and Delta and Omicron variant spike proteins were measured using pseudovirus neutralization assays. The geometric mean titers (GMTs) after the primary and booster immunizations were 12.09 and 61.48 for the wild-type strain, 11.67 and 40.33 for the Delta variant, and 8.51 and 29.31 for the Omicron variant, respectively. The GMTs against the wild-type strain declined gradually during the 12 months after the primary immunization, and were lower against the two variants. After implementing a booster immunization with a 6 month interval, the GMTs against the wild-type strain were higher than those obtained beyond the 7 month interval; however, the GMTs against the two variants were not statistically different across 3–12 month intervals. Overall, SARS-CoV-2 variants showed remarkable declines in immune persistence, especially against the Omicron variant. The booster administration interval could be shortened to 3 months in endemic areas of the Omicron variant, whereas an appropriate prolonging of the booster administration interval did not affect the booster immunization effect.

## 1. Introduction

COVID-19 continues to spread worldwide and is one of the most significant life-threatening infectious diseases in humans [1]. Governments around the world tried to stop the pandemic by mass vaccination. As of 30 September 2022, more than 356 COVID-19 vaccine candidates are currently in development. Moreover, 34 COVID-19 vaccines (including inactivated, protein subunit, adenovirus vector, and mRNA vaccines) are approved globally, and widespread vaccination programs are actively being implemented in over 200 countries/regions [2]. To date, three COVID-19 vaccines have been approved by the China Food and Drug Administration for human use: inactivated, viral vectored, and recombinant protein vaccines. In China, a total of 3.4 billion doses of COVID-19 vaccines have been administered as of 28 September 2022 [3], the vast majority of which were inactivated vaccines.

The hope is that the rapid development and deployment of COVID-19 vaccines against severe acute respiratory syndrome coronavirus 2 (SARS-CoV-2) might play a role to bring the COVID-19 pandemic under control [4]. Nevertheless, SARS-CoV-2 variants continue to emerge and spread worldwide. All kinds of vaccines are facing challenges, e.g., due to virus mutations. Currently, five variants of concern (VOCs), namely Alpha, Beta, Gamma, Delta, and Omicron, have been defined by the World Health Organization (WHO). As a result of specific mutations in their spike regions, these VOCs may escape immunity induced by prior infections or vaccines, thereby potentially causing a large number of breakthrough infections, either after natural infection or vaccination [5,6,7]. Additionally, immune protection against SARS-CoV2 variants decreases generally within 6–12 months following a primary infection or vaccination [8,9,10].

Facing the challenges posed by circulating VOCs and waning immune protection, booster vaccination has become a standard procedure. Booster vaccines ameliorate waning immunity and augment the breadth of immunity against SARS-CoV-2 VOCs [11]. Moreover, a preliminary evaluation has demonstrated that an increased level of antibodies was observed after a third CoronaVac dose eight months after the second dose. Furthermore, a third dose administered two months after the second dose induced slightly more elevated antibody titers than the primary two doses [12]. However, compared with other strains of SARS-CoV-2, Omicron is associated with a lower neutralization performance [13]. Whether different intervals of inactivated booster vaccines could lead to a better neutralization performance against Omicron remains unknown. 

Amid the ongoing efforts to vaccinate target populations, it is important to understand to what extent this resurgence is due to various factors, including: (1) the high infectiousness of the Delta and Omicron variants, (2) lower protection of the vaccine against these variants, and (3) decreasing levels of anti-SARS-CoV-2 antibodies against all strains in vaccinated individuals [14]. An optimal solution may be to administer a third dose of the vaccine after the second dose to enhance and prolong protection. Developing strategies to counter various SARS-CoV-2 variants should recognize two major considerations: (1) how long does vaccine-induced immunity last and (2) by how much immunogenicity can be improved by a booster. However, little is known about antibody attenuation during the 12 months after primary immunization and the immunogenic features of a COVID-19 inactivated vaccine booster dose at different time intervals. To answer these questions, we conducted a real-world study with two objectives. First, we sought to measure the attenuation degree of neutralizing antibodies (NAbs) against the SARS-CoV-2 wild-type strain, and Delta and Omicron variants one year after primary immunization. Second, we evaluated the immunogenicity of a COVID-19 inactivated booster vaccine at different intervals ranging from 3 to 12 months between the primary and booster doses.

## 2. Materials and Methods

### 2.1. Study Design

This cross-sectional post-marketing study of a COVID-19 inactivated vaccine was conducted in Beijing and comprises two parts. First, we assessed immune persistence by examining the NAbs against the SARS-CoV-2 wild-type strain, and Delta and Omicron variants among participants who had received a primary immunization 3–12 months prior to their enrollment in this study. Second, we assessed immunogenicity after the third booster dose was administered at different time intervals (3–12 months between the primary and booster immunizations).

### 2.2. Study Participants

Participants (N = 514) were included in this study based on the following criteria: (1) aged 18 years and above, (2) received two doses of COVID-19 inactivated vaccine at least three months prior to the study’s commencement, and (3) provided consent to participate in the study and undergo blood sampling. Participants were excluded based on the following criteria: (1) contraindications to vaccination, (2) previous serious diseases or certain healthy conditions, including autoimmune diseases or immunodeficiency/immunosuppression; serious chronic diseases; malignant tumors; serious nervous system disease or mental illness; individuals receiving immunosuppressive therapy, cytotoxic therapy, and corticosteroid inhalation one month prior to booster immunization; pregnancy, lactation, or planning to be pregnant; acute illness or an acute chronic disease attack seven days prior to the booster immunization, and (3) individuals who failed to follow-up and whose blood samples were not collected after the booster dose. The age median (P25, P75) was 35 (29, 43) years, with minimum and maximum ages of 19 and 62 years, respectively. The number of participants in the 18–29, 30–39, 40–49, and 50–62 age groups were 153, 183, 122, and 56, respectively. All participants provided informed consent voluntarily and free of charge prior to enrollment. The protocol of the clinical trial and the informed consent forms were approved by the Beijing Center for Disease Control and Prevention (BJCDC-- 2021-13). This trial was registered at ClinicalTrials.gov (NCT04962308) and was conducted in accordance with the requirements of the Good Clinical Practices of China and the International Conference on Harmonization.

### 2.3. Sample Collection

Venous blood samples were collected from all participants on the day of the booster immunization prior to inoculation. The participants were then vaccinated with a booster dose at different intervals ranging from 3 to 12 months, and venous blood samples were collected again 14–35 days after the booster dose. Briefly, all participants were divided into seven groups according to the different intervals between the second and third doses of the COVID-19 inactivated vaccine: a 3 month interval (n = 73), 4 month interval (n = 75), 5 month interval (n = 75), 6 month interval (n = 74), 7 month interval (n = 75), 8 month interval (n = 69), and 9–12 month interval (n = 73). Approximately 5 ml of venous blood was collected at each time point.

### 2.4. Cells and Plasmid

The 293T cells were obtained from the American Type Culture Collection (ATCC; VA, USA) and the 293T-hACE2 cells were purchased from Sino Biological Inc. (Beijing, China). The 293T cells were cultured at 37 °C in a humidified atmosphere with 5% CO_2_ in a high-glucose Dulbecco’s modified Eagle’s medium (HyClone, Utah(UT), USA) supplemented with 100 U/mL of penicillin-streptomycin solution (PS; Gibco, New YorK (NY), USA), 20 mM N-2-hydroxyethyl piperazine-N-2-ethane sulfonic acid (HEPES; New YorK (NY), Gibco), and 10% fetal bovine serum (ExCell Bio, Shanghai, China). Similarly, the 293T/hACE2 cells were cultured at 37 °C in a humidified atmosphere with 5% CO_2_ in a high-glucose Dulbecco’s modified Eagle’s medium (HyClone) supplemented with 100 U/mL PS (Gibco), 50 µg/mL hygromycin B (Invitrogen, Massachusetts (MA), USA), and 10% fetal bovine serum (ExCell). Trypsin-EDTA (0.25%; New YorK (NY), Gibco) was used to detach the cells for subculturing every 2–3 days.

The SARS-CoV-2 spike protein genes of the wild-type (Wuhan-Hu-1) strain (GenBank: MN908947), B.1.617.2 (full-length spike protein gene obtained from an epidemic in Beijing) and B.1.1.529 (GISAID: EPI_ISL_6640916) with a C-terminal 19 amino acid deletion were codon-optimized for Homo sapiens and cloned into the eukaryotic expression plasmid pcDNA3.1 (+) to generate the envelope expression plasmids pcDNA3.1-Wuhan-HU-1-S, pcDNA3.1-B.1.617.2-S, and pcDNA3.1-B.1.1.529-S, respectively. The lentiviral envelope and packaging plasmids pCD/NL-BH*DDD and luciferase plasmids pLenti CMV V5-LUC Blast (w567-1) were obtained from Addgene (Massachusetts (MA), USA).

### 2.5. Pseudovirus Preparation and Titration

Pseudoviruses with Wuhan-Hu-1, B.1.617.2, or B.1.1.529 spike proteins were prepared by co-transfecting 293T cells with pCD/NL-BH*DDD, pLenti CMV V5-LUC Blast (w567-1), and either pcDNA3.1-Wuhan-HU-1-S, pcDNA3.1-B.1.617.2-S, or pcDNA3.1-B.1.1.529-S. Briefly, 24 h prior to the transfection, 293T cells (7 × 10^5^ cells/ml) were inoculated into T-75 cell culture flasks and cultured at 37 °C in a humidified atmosphere with 5% CO_2_. The pre-plated cells were 70–90% confluent at the time of transfection, and the three plasmids (24 µg in total, mass ratio: 2:5:3) were co-transfected into 293T cells using Lipofectamine 3000 (Invitrogen). The supernatant was harvested 48 h post-transfection, centrifuged (1500× *g*, 10 min), filtered (0.45-μm pore size; Millipore, Darmstadt, Germany), aliquoted (1 mL/tube), and stored at −80 °C for further use.

Before pseudovirus titration, a single aliquot of the pseudotyped virus was removed from −80 °C to avoid repeated freezing and thawing. Subsequently, titration was carried out by serial dilution to infect the target 293T-hACE2 cells in 96-well plates. The initial dilution was repeated in four wells of a 96-well culture plate, followed by serial 5-fold dilutions (nine dilutions in total). The last column served as the control cell and did not contain a pseudovirus. Thereafter, 96-well plates were seeded with trypsin-treated 293T-hACE2 cells to a final concentration of 2.5 × 10^4^ cells/well. After 48 h of incubation at 37 °C in a humidified atmosphere with 5% CO_2_, the culture supernatant was gently aspirated to leave 100 μL in each well, after which 100 μL of Britelite Plus Reporter Gene Assay System (PerkinElmer, Massachusetts(MA), USA) was added to each well. Two minutes after incubation at a room temperature of approximately 20–22 °C, 150 μL of lysate was transferred to white solid 96-well plates for luminescence detection using a SpectraMax® iD5 microplate reader (Molecular Devices, California(CA), USA). The relative luminescence unit (RLU) values of the positive well were three-fold higher than that of the cell background. The 50% tissue culture infectious dose (TCID_50_) of the pseudotyped virus was calculated according to the Reed–Muench method [15].

### 2.6. Pseudovirus Neutralization Assay

The titers of the NAbs were quantified for Wuhan-Hu-1, B.1.617.2, and B.1.1.529 using the pseudovirus neutralization assay. First, 100 μL of serial three-fold diluted human serum (starting at 1:10) was incubated in 96-well plates with 50 μL of pseudovirus (64000 TCID_50_/mL) for 1 h at 37 °C. Thereafter, 293T-hACE2 cells were added (2.5 × 10^4^ cells/100 μL per well) and the plates were incubated at 37 °C in a humidified atmosphere with 5% CO_2_. Duplicate wells were analyzed for each sample. The cell control (CC), which contained only 293T-hACE2 cells, and the virus control (VC), which contained both the virus and 293T-hACE2 cells, were set up for each plate. After incubation for 48 h, chemiluminescence signals were detected via Molecular Devices SpectraMax® iD5 using the Britelite Plus Reporter Gene Assay System (PerkinElmer). The 50% inhibitory dose (ID_50_) was defined as the serum dilution at which the RLU was reduced by 50% compared to that of the VC wells after subtracting the background RLU in the CC wells. An ID_50_ value of ≥20.0 was considered positive.

### 2.7. Statistical Analysis

The immunogenicity endpoint was measured as the geometric mean titer (GMT) and seropositivity rate of the NAbs to the pseudoviruses of Wuhan-Hu-1, B.1.617.2, and B.1.1.529 before and after the third vaccine dose. SPSS (version 17.0, SPSS Inc., Chicago, IL, USA) was used for the statistical analyses. The enumeration data were analyzed by constituent ratios and 95% confidence intervals (CI), and the measurement data were analyzed by GMTs and the corresponding 95% CI. An analysis of variance (ANOVA) or a nonparametric test was used to compare the mean differences among the different groups, while Pearson’s χ^2^ test or Fisher’s exact test was used to compare the differences in the rates or constituent ratios among the different groups. When comparisons between more than two groups showed significant differences, pairwise comparisons were performed. Hypothesis testing was two-sided, and P-values of <0.05 were considered significant.

## 3. Results

### 3.1. Immune Persistence 3–12 Months after Two Primary COVID-19 Inactivated Vaccine Doses

**Figure 1:**
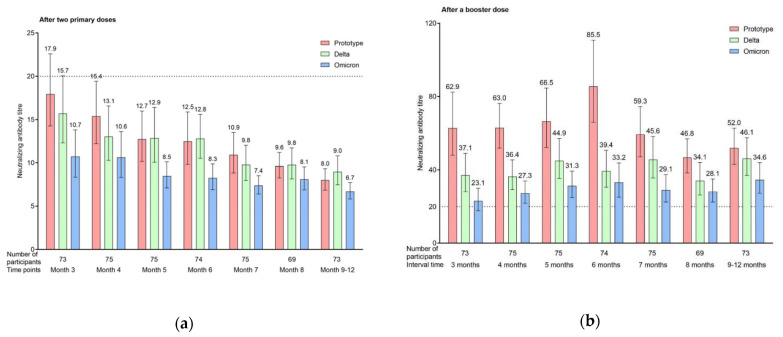
Neutralizing antibody titer against SARS-CoV-2 wild-type strain, and Delta and Omicron variants after primary and booster immunizations: (**a**) neutralizing antibody titer 3–12 months after two primary COVID-19 inactivated vaccine doses; (**b**) neutralizing antibody titer of a booster dose with different 3–12 month intervals.

**Figure 2:**
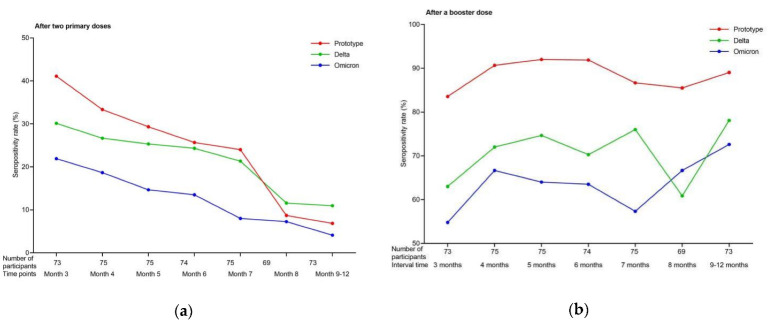
Seropositivity rate against SARS-CoV-2 wild-type strain, and Delta and Omicron variants after primary and booster immunizations: (**a**) seropositivity rate 3–12 months after two primary COVID-19 inactivated vaccine doses; (**b**) seropositivity rate of a booster dose with different 3–12 month intervals.

For the wild-type strain, the GMT of the NAbs was 17.95 (95% CI, 14.25–22.60) at 3 months after the primary immunization, with a seropositivity rate of 41.10%. At 4–7 months, the GMT gradually decreased to 15.40 (95% CI, 12.21–19.43; 4 months), 12.75 (95% CI, 10.16–16.00; 5 months), 12.50 (95% CI, 9.83–15.89; 6 months), and 10.93 (95% CI, 8.85–13.51; 7 months), and the seropositivity rate decreased to 33.33%, 29.33%, 25.68%, and 24.00%, respectively. At 8 and 9–12 months, the GMTs decreased to <10, the exact values being 9.62 (95% CI, 8.25–11.21; 8 months) and 8.00 (95% CI, 6.86–9.33; 9–12 months), and the seropositivity rates decreased to 8.70% and 6.85%, respectively. The GMT and seropositivity rates were significantly different at varying time points after the primary immunization in all groups (F = 6.657, P < 0.001; χ^2^ = 36.834, P < 0.001). Both the GMT and seropositivity rate at 3–4 months were higher than those after 7 months (P < 0.05) (Figure 1 and Figure 2).

The neutralization capacity against the variants was lower than that against the wild-type strain. During the 3–6 months, the GMT gradually decreased from 15.72 (95% CI, 12.31–20.07) to 12.81 (95% CI, 10.53–15.60) for the Delta variant, and the seropositivity rate ranged from 30.14% to 24.32%. At 7–12 months, the GMT decreased to <10, with the exact values being 9.79 (95% CI, 7.97–12.04; 7 months), 9.77 (95% CI, 8.15–12.29; 8 months), and 8.99 (95% CI, 7.46–10.83; 9–12 months) for the Delta variant, and the seropositivity rate declined to 21.33%, 11.59%, and 10.96% for 7, 8, and 9–12 months, respectively. In contrast, the GMT of the Omicron variant remained >10 during the 3–4 months and decreased from 10.73 (95% CI, 8.34–13.80; 3 months) to 10.64 (95% CI, 8.32–13.62; 4 months). From 5–12 months, the GMT of the Omicron variant decreased to <10, ranging from 6.71 (95% CI, 5.83–7.71) to 8.48 (95% CI, 7.12–10.12). The seropositivity rate for the Omicron variant ranged from 13.51% to 21.95% during the 3–6 months, and from 4.11% to 8.00% during the 7–12 months. The GMT and seropositivity rate of the two analyzed variants changed significantly until 12 months (Delta: F = 3.505, P = 0.002; χ^2^ = 14.186, P = 0.028. Omicron: F = 3.357, P = 0.003; χ^2^ = 16.572, P = 0.011). Both the GMT and seropositivity rates for the Delta and Omicron variants were higher at 3 months than those after 7 months (P < 0.05) (Figure 1 and Figure 2).

**Figure 3:**
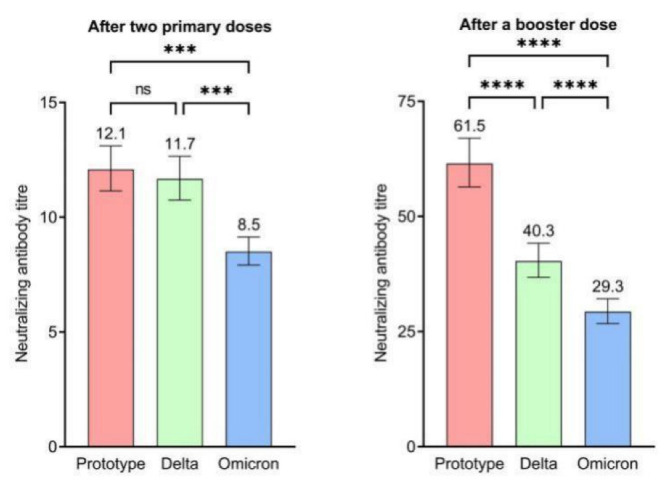
Neutralizing antibody titer against the SARS-CoV-2 wild-type strain, and Delta and Omicron variants after primary and booster immunizations (ns: not significant; ***: *p* < 0.001; ****: *p* < 0.0001).

In total, the respective GMT and seropositivity rates were 12.09 (95% CI, 11.15–13.11) and 24.32% for the wild-type strain, 11.67 (95% CI, 10.75–12.66) and 21.60% for the Delta variant, and 8.51 (95% CI, 7.92–9.15) and 12.65% for the Omicron variant. Furthermore, the GMT and seropositivity rates were the highest for the wild-type strain and the lowest for the Omicron variant (F = 23.170, P < 0.001; χ^2^ = 24.405, P < 0.001) (Figure 3).

**Figure 4:**
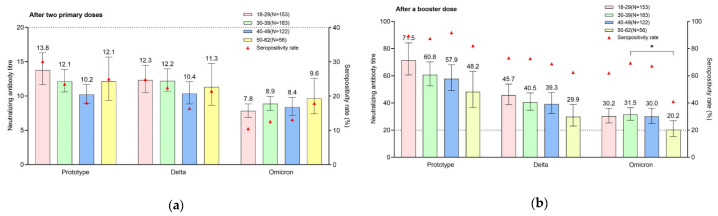
Neutralizing antibody titer against SARS-CoV-2 wild-type strain, and Delta and Omicron variants between different age groups: (**a**) neutralizing antibody titer after two primary COVID-19 inactivated vaccine doses; (**b**) neutralizing antibody titer after a booster dose (*: *p* < 0.05).

The analyses conducted in each age group after the administration of the two primary doses showed that the respective GMTs for the wild-type strain were 13.76 (95% CI, 11.63–16.29), 12.13 (95% CI, 10.59–13.89), 10.21 (95% CI, 8.93–11.67), and 12.12 (95% CI, 9.36–15.68), with seropositivity rates of 30.07%, 23.50%, 18.03%, and 25%, in the 18–29, 30–39, 40–49, and 50–62 age groups. The respective GMTs for the Delta variant were 12.32 (95% CI, 10.48–14.48), 12.20 (95% CI, 10.67–13.95), 10.35 (95% CI, 8.86–12.08), and 11.30 (95% CI, 8.66–14.74), with seropositivity rates of 24.84%, 22.40%, 16.39%, and 21.43% in the 18–29, 30–39, 40–49, and 50–62 age groups. The respective GMTs for the Omicron variant were 7.83 (95% CI, 6.91–8.86), 8.87 (95% CI, 7.91–9.96), 8.38 (95% CI, 7.16–9.81), and 9.64 (95% CI, 7.40–12.57), with seropositivity rates of 10.46%, 12.57%, 13.11%, and 17.86% in the 18–29, 30–39, 40–49, and 50–62 age groups. No significant difference in GMT and seropositivity rates for both the wild-type strain and Delta and Omicron variants was found between the different age groups (wild-type strain: F = 2.316, P = 0.075; χ^2^ = 5.446, P = 0.142. Delta: F = 0.985, P = 0.399; χ^2^ = 2.971, P = 0.396. Omicron: F = 1.111, P = 0.344; χ^2^ = 2.065, P = 0.559) (Figure 4).

### 3.2. Immunogenicity of a Booster Dose with Different 3–12 Month Intervals

After the third vaccine dose was administered, the respective GMT and seropositivity rates of the NAbs against the wild-type strain were 62.91 (95% CI, 47.98–82.45) and 83.56% at the 3 month interval, 62.98 (95% CI, 51.95–76.35) and 90.67% at the 4 month interval, 66.51 (95% CI, 52.24–84.68) and 92.00% at the 5 month interval, 85.55 (95% CI, 66.01–110.84) and 91.89% at the 6 month interval, 59.32 (95% CI, 47.16–74.61) and 86.67% at the 7 month interval, 46.76 (95% CI, 38.36–56.99) and 85.51% at the 8 month interval, and 51.96 (95% CI, 43.00–62.81) and 89.04% at the 9–12 month interval. The highest GMTs were observed at the 6 month interval after the booster dose. Furthermore, the GMT at the 6 month interval was higher than that at intervals >7 months (F = 2.757, P = 0.012), whereas the differences in the seropositivity rate were not significant at the 3–12 month intervals (χ^2^ = 4.718, P = 0.580). Compared to the NAbs level before the booster immunization, the GMT of the NAbs increased 3.5–6.8-fold across the 3–12-month intervals (Figure 1 and Figure 2).

After the third vaccine dose was administered, the neutralization capacities against the variants were lower than those against the wild-type strain. The GMT ranged from 34.08 (95% CI, 26.35–44.09) to 46.13 (95% CI, 36.95–57.58) and the seropositivity rate ranged from 60.87% to 78.08% for the Delta variant. Additionally, the GMT ranged from 23.07 (95% CI, 17.73–30.03) to 34.63 (95% CI, 27.29–43.95) and the seropositivity rate ranged from 54.79% to 72.60% for the Omicron variant. However, no significant differences were observed at the 3–12 month intervals for Delta (F = 0.982, P = 0.437; χ^2^ = 8.893, P = 0.180) or Omicron (F = 1.232, P = 0.288; χ^2^ = 6.865, P = 0.334) variants. The GMT of the NAbs against the two analyzed variants increased 2.2–5.2-fold across the 3–12 month intervals compared to that before the booster immunization (Figure 1 and Figure 2).

In total, the respective GMT and seropositivity rates after the third dose were 61.48 (95% CI, 56.42–66.99) and 88.52% for the wild-type strain, 40.33 (95% CI, 36.79–44.20) and 70.82% for the Delta variant, and 29.31 (95% CI, 26.75–32.11) and 63.62% for the Omicron variant. Furthermore, the GMT and seropositivity rates were the highest for the wild-type strain and the lowest for the Omicron variant (F = 64.946, P < 0.001; χ^2^ = 88.460, P < 0.001). Compared to the NAbs level before the booster immunization, the GMT of the NAbs increased 5.1-fold for the wild-type strain, 3.5-fold for the Delta variant, and 3.4-fold for the Omicron variant across the 3–12 month intervals (Figure 3).

The analyses conducted in each age group after the booster dose administration showed that the respective GMTs for the wild-type strain were 71.47 (95% CI, 60.65–84.22), 60.79 (95% CI, 52.58–70.29), 57.86 (95% CI, 49.17–68.09), and 48.24 (95% CI, 36.78–63.28), with seropositivity rates of 89.54%, 87.43%, 91.80%, and 82.14% in the 18–29, 30–39, 40–49, and 50–62 age groups. The respective GMTs for the Delta variant were 45.72 (95% CI, 38.76–53.94), 40.50 (95% CI, 34.67–47.33), 39.26 (95% CI, 32.27–47.74), and 29.90 (95% CI, 22.97–38.92), with seropositivity rates of 73.20%, 72.68%, 68.85%, and 62.50% in the 18–29, 30–39, 40–49, and 50–62 age groups. The respective GMTs for the Omicron variant were 30.19 (95% CI, 25.24–36.10), 31.52 (95% CI, 27.24–36.47), 30.04 (95% CI, 25.03–36.06), and 20.20 (95% CI, 15.15–26.92), with seropositivity rates of 62.09%, 69.40%, 67.21%, and 41.07% in the 18–29, 30–39, 40–49, and 50–62 age groups. Although the GMT and seropositivity rates for the wild-type strain and Delta and Omicron variants in the 50–62 age group were lower than those in the other age groups, only the difference for the Omicron variant was significant (wild-type strain: F = 2.472, P = 0.061; χ^2^ = 3.906, P = 0.272. Delta: F = 2.251, P = 0.082; χ^2^ = 2.830, P = 0.419. Omicron: F = 2.706, P = 0.045; χ^2^ = 15.777, P = 0.001) (Figure 4).

## 4. Discussion

The present study is a large-scale and long-term prospective cohort study on the antibody persistence and secondary immune response to the COVID-19 vaccine. Using pseudovirus neutralization assays for SARS-CoV-2, the immunogenicity of 514 participants was evaluated 3–12 months after primary immunization, and 14–35 days after the booster immunization in seven intervals (3, 4, 5, 6, 7, 8, and 9–12 months elapsed between the primary and booster doses). A previous study has shown that high levels of NAbs develop approximately 14–28 days following the initial COVID-19 immunization, after which the antibody level gradually decreases over time, even decreasing significantly after 6–8 months [16]. Moreover, the magnitude of this decline varies between different vaccines. This post-vaccination phenomenon of decreasing protective antibody levels also occurs with numerous other vaccines. Particularly, published studies on vaccines against measles, mumps, and rubella have shown small annual decreases by 5–10% in NAb levels [17,18]. Additionally, after six months of primary vaccination, CoronaVac’s phase 2 clinical trial found that 80% of participants’ NAb titers decreased below the seropositivity cutoff of eight [12]. However, since no blood specimens were collected between 28 days and 6 months after the second dose, it was impossible to determine when the seropositive status changed to a seronegative status. There is also an ongoing study regarding whether a booster dose can extend the duration and spectrum of activity against emerging viral variants beyond six months. Based on a model of NAb titers decaying over 250 days after immunization, significant protection against SARS-CoV-2 infection is predicted, and severe infections should be largely prevented. Additionally, despite limited data on the duration of vaccine-induced immunity, the decay of vaccine-induced neutralization is similar to that observed following natural infection with SARS-CoV-2 [19]. Therefore, infection-induced immune protection may also wane with time as neutralization levels decline [20].

Our study reported the duration of the immune response following a two-dose primary vaccination with a COVID-19 inactivated vaccine at different time points during one year and the immunogenicity of a booster dose at different intervals of 3–12 months. The results of this study showed that the highest GMT and seropositivity rate against the wild-type strain and Delta and Omicron variants was at three months after primary immunization. Thereafter, the GMT and seropositivity rate gradually declined until 12 months after the second vaccination dose. Furthermore, 7–12 months after the second vaccination dose, the GMTs of the NAbs and the seropositivity rates declined further. The neutralization capacity for the Omicron variant was extremely low, even at three months after primary immunization. This is consistent with the antibody persistence results of other COVID-19 vaccines after full inoculation. Previous studies have shown that the high peak antibody responses against live wild-type and pseudo viruses decline sharply until six months and further decline until eight months after the completion of primary immunization [16]. Within six months after CoronaVac vaccination, NAb titers decline 7.3-fold, and NAb titers of some SARS-CoV-2 variants (e.g., B.1.351 Beta and P1 Gamma) circulating are reduced 8–10-fold. With its weaker immunogenic effect, CoronaVac vaccines are less likely to provide prolonged protection [21]. Therefore, in terms of immunogenicity, the two-dose primary immunization only achieved a low degree of neutralization and protective effect [22]. Further, there is an increasing attention on a wider consideration of the different facets of vaccine-induced protective immunity and its durability, which may be related to immune memory rather than antibody titers. The initial serum antibody wanes in the absence of ongoing antigenic stimulation, but that B and T cell memory will have been primed for a speedy, protective response upon a repeat encounter. Our previous research on the functional CD4+ and CD8+ memory T cells response in participants administrated by two doses of COVID-19 inactivated vaccine suggested that the structural integrity of whole SARS-CoV-2 of inactivated vaccine might be the key to elicit antiviral CD8+ memory T-cell responses, and Th1 dominated the immune response pathway rather than the traditional Th2 [23].

With regard to escape from vaccine-induced immunity, the Omicron variant showed different decreases in neutralizing activity in the serum samples obtained from vaccinated persons, which was lower by a factor of 1.6 for the inactivated vaccine (BBIBP-CorV), 6.5 for BNT162b2 (BioNTech/Pfizer) mRNA vaccine and 8.6 for mRNA-1273 (Moderna) vaccine, but it was lower by a factor of up to 86, including complete immune escape, for the AZD1222 (AstraZeneca) viral vector vaccine [24]. Compared with mRNA, recombinant protein, or viral vector vaccines involving only receptor-binding domain (RBD) or spike(S) protein, an advantage of inactivated COVID-19 vaccines may be that they also contain conserved SARS-CoV-2 antigens in addition to S protein [25]. This means that more epitopes, especially those conserved epitopes in proteins other than S protein, are also engaged in T-cell responses induced by inactivated COVID-19 vaccines [26,27]. Therefore, in a head-to-head comparison, CoronaVac elicited higher structural protein-specific CD4+ and CD8+ T-cell responses than Pfizer BNT162b2, due to the presence of additional nucleocapsid (N) and envelope (E) proteins [21].

When NAbs decline to a certain extent following primary immunization, a booster immunization may be required within a year to improve immune responses. A phase 2 clinical trial of the CoronaVac vaccine showed that a third dose administered at six or eight months after the second dose rapidly and markedly increased the NAbs titers: the antibody level was 10–30-fold higher after 6 months than that before the booster immunization, and was approximately 7-fold higher after 8 months than that on day 28 after the second immunization. The CoronaVac-induced neutralization capacity against the original SARS-CoV-2 at four weeks after a third dose is approximately 60% higher than that after only two doses [12]. Moreover, a previous study showed that booster immunization has good cross-neutralization capacities for a variety of mutant strains, thereby exerting a stronger and broader protective effect against COVID-19. Therefore, it is necessary to administer a third dose to obtain the desired effect against Wuhan-Hu-1 or the variants after the two vaccine doses were administered [28,29]. The third dose could either be a booster immunization or part of the primary vaccination schedule. Obtaining the best immunogenicity requires a determining of the optimal interval between the second and third doses. A study on the immunogenicity of a shorter interval between the primary and booster immunizations showed that a third dose administered 28 days after the second dose resulted in much lower antibody levels than one administered 6 months or later. As observed in patients with COVID-19, memory B-cells against SARS-CoV-2 are more abundant at six months following the onset of symptoms than at one month.

In this study, the interval between the primary and booster immunizations ranged from 3 to 12 months. The results showed that GMT rapidly increased after the booster immunization for the wild-type strain and Delta and Omicron variants. In total, the level of NAbs after the booster immunization was 3–5-fold higher than that before booster immunization. Moreover, both the GMT and seropositivity rates after booster immunization against the wild-type strain were higher than those against the Delta and Omicron variants. Although the GMT after the booster dose against the wild-type strain was higher at the 6 month interval, there were no significant differences in GMTs and seropositivity rates against the two variants between 3 and 12 month intervals.

Our study had several limitations. First, the participants were primarily healthy adults and did not sufficiently represent older individuals with underlying medical conditions. Second, the sample size in the groups with longer intervals between the primary and booster immunizations was relatively small. Therefore, we combined the data of participants who were vaccinated with the booster dose at intervals of more than 9 months. Third, the polyclonal immune response includes T- and B-cell responses of the adaptive immune response. The innate immune responses are not included in the adaptive polyclonal responses. Although there have been previous researches on the role of mucosal immunity in preventing infection [30,31], the mucosal immune responses of the lung after vaccination and how the compartmentalization (lung vs blood) effects the efficacy of the present vaccination have not been addressed in this study, along with various limiting factors such as vaccine type, the source of participants, and sample type. 

## 5. Conclusions

The findings of this study are instructive for the adjustment of booster immunization strategies. To rapidly elevate the antibody titers, a booster dose should be administered six months after the completion of a primary series; this interval could be shortened to three months in outbreaks and in endemic areas of the Omicron variant. However, since a prolongation of the interval between the primary and booster doses did not affect the booster immunization effect, an appropriate prolongation of this interval could be applied in non-endemic regions or when faced with a shortage of COVID-19 vaccines. Finally, due to the relatively low antibody response observed against the Omicron variant, multivalent or Omicron-specific vaccines are urgently needed to maximize the impact of the vaccination program with the primary aim of reducing mortality and protecting medical institutions against the surge in hospitalizations. Future vaccine strategies in the face of emerging variants of concern need more research on a good early warning for emerging VOC, optimal immunization schedules in terms of vaccine design, the number of doses, dosing interval, and approaches to achieve safe, durable vaccine immunity and generate truly variant cross-protective immunity.

## Data Availability

The data that support the findings of this study are available from the corresponding author [wj81732@hotmail.com (J.W.)], upon reasonable request.
